# Impact of ChatGPT-assisted personalized learning on teaching acute abdomen to undergraduate medical students: A randomized crossover study

**DOI:** 10.12669/pjms.42.8.14827

**Published:** 2026-08

**Authors:** Maria Ilyas, Rehan Ahmed Khan, Rahila Yasmeen, Zainab Kamal, Noor ul Ain

**Affiliations:** 1 Maria Ilyas, MHPE Senior Lecturer Pharmacology, Watim Medical and Dental College Rawat, Rawalpindi, Pakistan; 2 Rehan Ahmed Khan, FCPS, PhD HOD Surgery & Dean RIA, Riphah International University, Islamabad, Pakistan; 3 Rahila Yasmeen, PhD-HPE Dean RARE, Director MHPE Program, Riphah International University, Islamabad, Pakistan; 4 Zainab Kamal, MHPE Research Officer Pakistan Armed Forces Medical Journal, Army Medical College, Rawalpindi, Pakistan; 5 Noor ul Ain, MHPE Assistant Professor Medical Education/Assistant Director RIA, Islamic International Medical College, Riphah International University, Islamabad, Pakistan

**Keywords:** Academic Performance, Adaptive Learning, Artificial Intelligence, Medical Education, Personalized Learning Pathways

## Abstract

**Background &Objective:**

The application of Artificial Intelligence (AI) in medical education has emerged as a promising avenue for personalized learning experiences to the individual needs of medical students. This study investigated the impact of AI-driven personalized learning pathways on the academic performance of medical students in acute abdomen topic, comparing against traditional learning method.

**Methodology:**

This study used a randomized controlled crossover trial conducted from February 2024 to July 2024 among fourth year one hundred undergraduate medical students at Islamic International Medical College, Riphah International University, Pakistan. In this study, students enrolled in a general surgery course were randomly assigned to experimental group and control group following a pre-test. The experimental group engaged with AI-driven personalized learning pathways, powered by ChatGPT-4, the control group utilized conventional educational resources. Both groups completed a post-test to assess the effects of their respective learning interventions. Statistical analyses, including descriptive statistics, independent-samples t-tests and paired-samples t-tests were conducted using SPSS.

**Results:**

The pre-test scores of the experimental (M = 17.12, SD = 6.99) and control groups (M = 18.64, SD = 6.91) did not differ significantly (p = 0.277). Post-intervention, the experimental group showed a statistically significant improvement (M = 22.8, SD = 5.11) compared to the control group (M = 19.24, SD = 7.11), with a p-value of .005 and a moderate effect size (Cohen’s d = 0.58). This suggests that AI-driven personalized learning pathways had a positive and measurable impact on the students’ academic performance in the experimental group.

**Conclusion:**

AI-driven personalized learning pathways can enhance the academic performance of medical students, particularly in subject of surgery. Future research should explore the long-term effects of personalized AI-powered educational interventions.

## INTRODUCTION

Medical education is increasingly incorporating Artificial intelligence (AI) into teaching and learning to support more learner-centered educational approaches.^1^ This incorporation resulted in application of deep learning (DL), a subset of machine learning (ML) that enables systems to learn and draw insights from big datasets.^2^ Its algorithms mimic the neural networks of the human brain, analyze large datasets, identify patterns that help in customizing educational content to suit individual learners’ needs.^2^-^4^ Conventional teaching and learning often follow a uniform model that may not address differences in students’ prior knowledge, pace of learning and individual support needs. As a result, there is growing interest in educational strategies that can provide more personalized learning experiences and improve academic outcomes in complex medical subjects.^3^

AI-enabled educational tools have shown potential to enhance personalization by offering tailored explanations, formative questioning, and feedback based on learner needs.^5^,^6^ In higher education such approaches have been explored to support adaptive learning and improve students’ engagement.^7^ In medical education, this potential is relevant because students need to integrate the knowledge with clinical reasoning in a limited time. However, despite growing interest in AI applications in healthcare and education, evidence regarding the educational impact of AI-assisted personalized learning in undergraduate medical training remains limited.

One of the challenges in medical education today is the widespread reliance on the “one-size-fits-all” approach, that fails to meet the diverse learning needs of students.^8^ AI-enabled educational tools, including generative AI platforms, may support more personalized learning (PLPs) through tailored explanations, formative questioning, and feedback.^9^ These systems can track and analyze learning behaviors, dynamically adjust content and provide feedback that optimizes the student’s learning experience.^10^–^12^ Medical education, particularly in subjects like general surgery and clinical decision-making, requires flexible and adaptive learning methods to accommodate the varied learning styles and needs of students.^13^ Acute abdomen is a clinically important and cognitively demanding topic for undergraduate medical students because it requires prompt recognition of symptoms, understanding of differential diagnoses, and appropriate interpretation of management principles.^12^ Teaching this topic therefore benefits from instructional approaches that allow clarification of misconceptions, reinforcement of key concepts, and opportunities for self-paced learning.

ChatGPT-4 is a conversational AI tool that can support learning through structured text-based interaction, including individualized explanations, case-based discussion, and practice questions. When used within defined curricular objectives and faculty oversight, it may serve as a useful adjunct to conventional learning resources. Nevertheless, relatively few controlled studies have examined whether ChatGPT-assisted personalized learning can improve short-term academic performance in focused areas of undergraduate medical education.^11^,^13^,^14^ Research in this area is often limited to traditional e-learning platforms or simulation-based methods, leaving a significant gap in understanding how AI-powered tools can specifically improve students’ comprehension of medical topics.

Therefore, this study aimed to compare the ChatGPT-assisted PLPs with traditional learning methods in teaching acute abdomen in general surgery course to undergraduate medical students. The primary objective was to evaluate short-term topic-specific academic performance, while the secondary objective was to assess students’ satisfaction with the AI-assisted learning experience.

## METHODOLOGY

This study used a randomized controlled crossover trial conducted among from February to July 2024 among fourth year undergraduate medical students at Islamic International Medical College, Riphah International University, Pakistan. Participants were randomly allocated to two groups (Group-A and Group-B). During Phase-1, Group-A received AI-assisted learning using ChatGPT-4, while Group-B received conventional teaching. In Phase-2, the interventions were crossed over.^15^

A priori sample size estimation using G*Power indicated a required sample of 107 participants for detection of a moderate effect at α=0.05 and power=0.95.^16^ After informed consent, one hundred students were enrolled and randomized into two groups (50 each). Students who declined participation were excluded.

### Participants:

The study included fourth-year undergraduate medical students enrolled in the general surgery course, focusing on the “Acute abdomen” topic. Students with declared learning disabilities or significant mental health conditions were excluded from this initial efficacy study to reduce heterogeneity in learning support needs and because the intervention had not yet been evaluated within a framework that included tailored accommodations. These exclusions were based on self-report and institutional disclosure rather than formal diagnostic screening. Baseline technology-use survey showed no prior use of AI-assisted personalized learning platforms. Stratified block randomization was used to ensure balance in gender and baseline pre-test scores between groups.^17^ No formal washout period was introduced between phases because the intervention involved topic-specific knowledge acquisition, and learned educational content could not be practically or ethically withdrawn.

### Randomization Process:

To balance key covariates between the experimental and control groups, gender and pre test scores were used as stratification factors during the randomization process. A block randomization method (block size = 4) was used to allocate students to the groups. A computer-generated randomization sequence was sealed in opaque envelopes and managed by an independent third-party to ensure unbiased group assignment. The randomization process resulted in balanced groups for gender and baseline scores, with no participant dropouts during the study.

### Intervention process:

The control group used traditional learning methods, including standardized surgical textbooks (e.g., Bailey & Love’s Short Practice of Surgery), lecture notes, and PowerPoint presentations provided during routine teaching sessions. The experimental group received AI-assisted personalized learning support using ChatGPT-4 (OpenAI). The AI system was used as an interactive educational tool to supplement conventional learning materials related to the topic of acute abdomen.

Structured prompts were designed and standardized by the research team prior to the intervention. These prompts guided ChatGPT-4 to generate explanations of clinical concepts, case-based discussions, and practice questions aligned with the learning objectives of the module. All participants in the intervention group used the same prompt framework to ensure consistency.

Students interacted with the AI system through text-based queries and received explanations, summaries, and additional examples to clarify key surgical concepts. The system also suggested supplementary learning resources, such as guideline summaries, diagrams, and educational materials from a faculty-curated list. To ensure academic accuracy, faculty members periodically reviewed AI-generated responses and advised students to verify important clinical information using standard textbooks and lecture materials.

Knowledge acquisition was assessed using blueprinted MCQs aligned with the learning objectives of the acute abdomen module. Pre-test (20 MCQs) and post-test (20 MCQs) assessments were constructed from the same question pool, but different items were used at each testing point. This ensured comparable sampling of the content domain while minimizing recall bias associated with repeated exposure to identical questions.

Students completed a form detailing their study habits and learning preferences, which helped the AI tailor the learning pathways. Both groups engaged in their respective learning methods for two hours. Afterward, both groups took a post-test to measure their learning progress. Following the initial comparison, the groups switched learning methods: the control group received PLPs, and the experimental group returned to traditional methods. After another hour of study, both groups completed a final post-test. The results were again analyzed to assess any performance differences based. Assessment procedures were standardized across groups. Both groups were tested under the same conditions using blueprinted MCQ-based assessments with comparable academic standards. Instrument and performance biases were addressed by ensuring equal academic standards in both tests and stratifying students into high, moderate, and low achievers for balanced group assignments. A post-intervention satisfaction survey assessed perceived accessibility, engagement, and usefulness of the AI platform

A pilot study with 10 students was conducted to refine study procedures and improve clarity of the intervention process. The primary goal was to evaluate the usability of the form and ensure that students could effectively engage with the AI-driven PLPs. Based on feedback from the pilot study, minor adjustments were made to improve clarity and functionality.

Data analysis was performed using SPSS version 26. Descriptive statistics were calculated to summarize the performance of students. Comparative analyses were conducted using independent-samples t-tests to compare performance outcomes. Additionally, paired samples t-tests were used to compare the pretest and posttest scores within each group. The gain in scores was calculated, and effect sizes (Cohen’s d) were computed to assess the magnitude of the differences between groups. The satisfaction survey results were analyzed to provide qualitative insights into the user experience.

### Ethical consideration:

The Institutional Review Board (IRB) (Riphah/IIMC/24/1016; dated February 12, 2024) granted ethical clearance. Every participant gave consent, privacy and anonymity were maintained. The AI tool was used only for educational support within a defined course context. No personal health data were entered into the system. Students were informed that AI-generated educational content could contain inaccuracies and were instructed to verify key information using faculty-approved resources.

## RESULTS

A total of one hundred students were included in the study, with fifty students assigned to the experimental group and 50 to the control group. Baseline characteristics, including age, gender, and pre-test scores, were comparable between the two groups with no statistically significant differences observed.

### Baseline data of the participants:

Following the intervention, the experimental group showed higher post-test scores (M = 22.8, SD = 5.11) than the control group (M = 19.24, SD = 7.11). This difference was statistically significant (p = 0.005), with a moderate effect size (Cohen’s d = 0.58), suggesting that ChatGPT-assisted personalized learning was associated with improved short-term academic performance. Table-I.

**Table-I:** Baseline data of the participants.

Indicator	Experimental Group (n = 50)	Control Group (n = 50)	Statistic	p-value	95% Confidence Interval
Age (years)	21.10 ± 1.12	21.12 ± 1.13	t = 0.09	0.923	-
Gender (male/female)	24/26	25/25	χ² = 0.04	0.846	-
Pre-test score (points)	17.12 ± 6.98	18.64 ± 6.91	t(98) = 1.094	0.277	-1.24 to 4.28

### Comparison of Scores in two groups:

**Fig.1:**
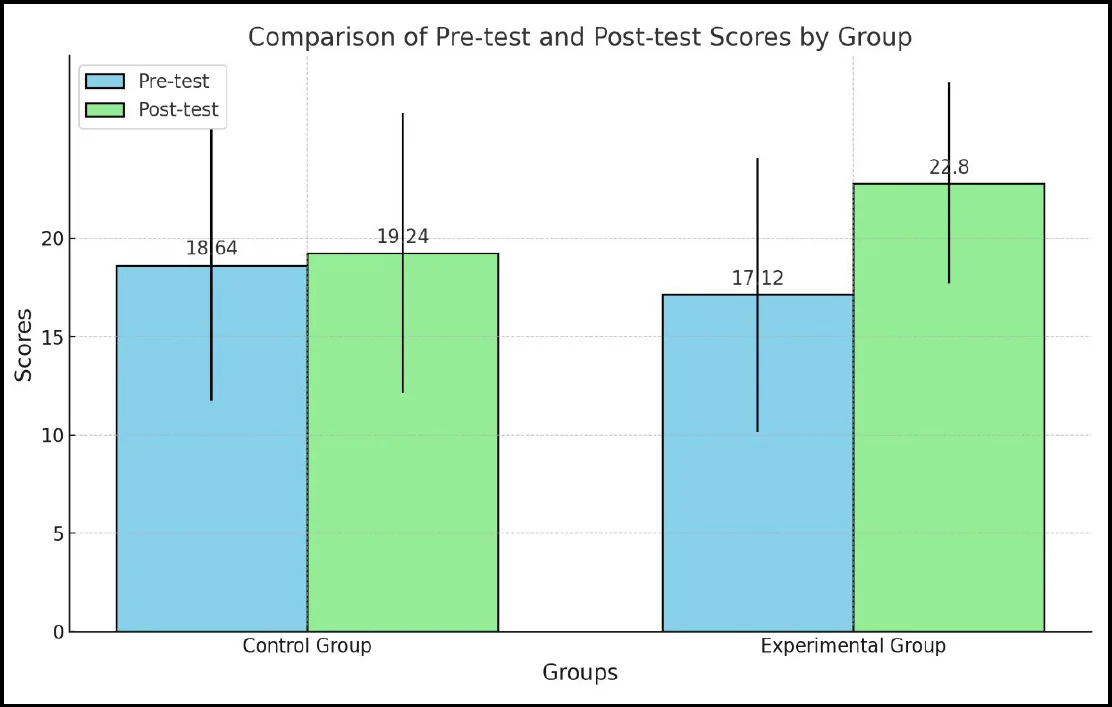
Comparison of Scores in two groups (Bar chart visualizing the pre-test and post-test scores for both the Control and Experimental groups. The bars represent the average scores for each group at each stage, with error bars indicating the standard deviations. This chart effectively highlights the difference in performance between the groups and the change in scores from pre-test to post-test).

While the control group exhibited just a slight improvement (mean change = 0.14 ± 0.41), the experimental group showed a significant increase from the pretest to the posttest (mean change = 4.16 ± 1.8). This difference highlighted the efficiency of AI-powered PLPs in improving students’ academic performance (Table-II). Fig.1

**Table-II:** Calculation of Gain from Pretest to Posttest Scores (n = 100).

Group	n	Pretest Mean ± SD	Posttest Mean ± SD	Change (Post–Pre)
Control Group	50	18.64 ± 6.91	19.24 ± 7.11	0.6±0.2
Experimental Group	50	17.12 ± 6.99	22.80 ± 5.11	5.6±1.3

### Calculation of Gain from Pretest to Posttest Scores (n = 100):

During the crossover phase, the experimental group’s mean score decreased slightly after switching from ChatGPT-assisted personalized learning to conventional learning methods, from 22.80 ± 5.11 to 21.66 ± 4.42; however, this change was not statistically significant (t(49) = 1.347, p = 0.184). The control group’s mean score increased from 19.24 ± 7.11 to 21.14 ± 4.62 after crossover to ChatGPT-assisted personalized learning, but this change did not reach statistical significance (t(49) = -1.75, p = 0.087). (Table-III).

**Table-III:** Crossover Analysis - Experimental and Control Groups.

Group	n	Posttest 1 Mean ± SD	Posttest 2 Mean ± SD	t(49)	p-value
Experimental Group	50	22.80 ± 5.11	21.66 ± 4.42	1.347	0.184
Control Group	50	19.24 ± 7.11	21.14 ± 4.62	-1.75	0.087

### Crossover Analysis - Experimental and Control Groups:

Following crossover, post-test 2 scores were similar in the experimental group (M = 21.66, SD = 4.42) and control group (M = 21.14, SD = 4.62), with no statistically significant difference (t(98) = 0.575, p = 0.567, Cohen’s d = 0.115). These findings are shown in Table IV.

**Table-IV:** Crossover Comparison of Posttest Scores after Crossover (n = 100).

Group	Mean	SD	t(98)	P-value	Cohen’s d	95% Confidence Interval	
						LL	UL
Control Group	21.14	4.62	0.575	0.567	0.115	-1.2753	2.31533
Experimental Group	21.66	4.42					

### Crossover Comparison of Posttest Scores after Crossover (n = 100):

The participants expressed satisfaction with AI-powered PLPs. The majority gave the platform an efficacy rating of “Excellent” or “Good,” highlighting its capacity to pinpoint both strengths and shortcomings and provide customized comments. Table-IV The importance of flexible timetables and real-time progress monitoring in improving motivation and content retention was emphasized by open-ended comments. These results highlight the potential of AI-driven PLPs to improve the surgery concepts in medical education by raising the academic achievements, student involvement, motivation, and satisfaction.

## DISCUSSION

The findings of this study provide evidence that ChatGPT-assisted personalized learning was associated with improved short-term academic performance in undergraduate medical students studying acute abdomen. The substantial improvement in academic performance observed in the experimental group supports recent studies that have demonstrated the positive impact of tailored learning tools on student outcomes.^5^,^13^,^18^ Specifically, our research contributes to the growing body of literature by showing that AI-driven platforms can provide personalized learning plans and feedback.

The comparatively smaller gain observed in the control group may reflect the limitations of conventional one-size-fits-all learning approaches in addressing individual differences. This aligns with the work of Tong et al. (2022), who suggested that generic, one-size-fits-all teaching strategies often fail to meet the diverse needs of learners.^10^ In contrast, AI-based personalized learning approaches appear to offer a more effective solution, as demonstrated by the larger improvements in the experimental group.

One of the most intriguing findings of this study was the crossover phase results. Although post-test 2 scores were similar between groups after crossover, the study did not include a formal washout period, and carryover effects cannot be excluded. In educational interventions involving knowledge acquisition, prior learning cannot be practically withdrawn between phases. Therefore, the crossover phase should be regarded as exploration and not as evidence of durable or long-term intervention effects. This aligns with the research by Martin et al. & Trang et al. (2018),^19^ who suggested that AI tools have the potential to foster lasting learning improvements and boost self-confidence among students, even after they transition back to conventional methods.

Unlike other studies focusing on digital interventions, this research offered deeper insights into the effects of ChatGPT platform on complex, content-heavy subjects like medicine.^20^,^21^ Studies by Sajja et al.^11^ (2024) and others have primarily examined the role of AI in general education or e-learning, but our study provides empirical evidence of AI’s specific utility in medical education, where subjects such as surgery require not only theoretical knowledge but also practical, decision-making skills.

Moreover, Student satisfaction findings were generally positive, with participants reporting favorable perceptions of the platform’s accessibility, engagement, and usefulness.^12^ Students appreciated the tailored learning experience and the immediate feedback provided by the AI platform, reinforcing the value of real-time, adaptive learning. However, this study stands out by demonstrating that the ability of AI tools to offer customized analytics and feedback is crucial not just for short-term engagement, but for sustaining academic motivation in the long run.

This study authors provided valuable insights into the power of AI-driven PLPs to personalize the learning experience for medical students. The results demonstrate that AI technologies, when integrated thoughtfully into the curriculum, can lead to substantial improvements in academic performance and student engagement. As digital tools continue to play a larger role in education, particularly in fields requiring complex, specialized knowledge like medicine, further research is needed to explore the long-term effects of such interventions and their broader applicability across diverse educational contexts.

### Limitations:

It was conducted at a single institution on one focused surgical topic, which limits generalizability. Students with declared learning disabilities or significant mental health conditions were excluded, which may have introduced selection bias and reduced inclusiveness. No formal washout period was used between crossover phases; therefore, carryover effects cannot be excluded. In addition, the short duration of the intervention did not permit evaluation of long-term retention or sustained educational impact.

## CONCLUSION

ChatGPT-assisted PLPs are associated with improved short-term academic performance in undergraduate medical students studying acute abdomen. The findings support the objective of exploring AI’s potential to improve learning outcomes by providing individualized learning experiences that adapt to each student’s needs. Future research should focus on exploring scalable models for AI integration and address the challenges to maximize its potential across medical institutions.

### Future suggestions:

Future studies should include larger and more diverse student populations, broader topic coverage, longer follow-up, and analytical approaches that formally account for sequence and period effects in crossover designs

### Author’s Contribution:

**MI:** Conceived, designed, literature searched and did the data analysis and is responsible for integrity of research.

**RAK, RY, ZK and NA:** data collection, data analysis, and interpretation.

**NA:** Did the critical review of the paper.

All authors have read and approved the final manuscript.
